# Differentiation Induction and Proliferation Inhibition by A Cell-Free Approach for Delivery of Exogenous miRNAs to Neuroblastoma Cells Using Mesenchymal Stem Cells

**DOI:** 10.22074/cellj.2021.6928

**Published:** 2020-04-22

**Authors:** Samaneh Sharif, Mohammad Hossein Ghahremani, Masoud Soleimani

**Affiliations:** 1 Department of Molecular Medicine, School of Advanced Technologies in Medicine, Tehran University of Medical Sciences, Tehran, Iran; 2 Medical Genetics Research Center, Mashhad University of Medical Sciences, Mashhad, Iran; 3 Department of Toxicology and Pharmacology, Faculty of Pharmacy, Tehran University of Medical Sciences, Tehran, Iran; 4 Department of Haematology, Tarbiat Modares University, Tehran, Iran

**Keywords:** Differentiation, Exosome, Mesenchymal Stem Cells, MiR-124, Neuroblastoma

## Abstract

**Objective:**

Neuroblastoma (NB) is one of the frequently observed malignant solid tumors of childhood and infancy, accounting for 15% of pediatric cancer deaths. Recently, the approach of differentiation therapy has shown considerable promise in effective treatment of NB patients. MiR-124 belongs to the nervous system-specific miRNAs that is increased during neuronal differentiation and may be one of the potential therapeutic targets for the treatment of NB. However, despite its well-established therapeutic potential, its efficient delivery to the targeted tumor cells is a challenging task. Mesenchymal stem cells (MSCs) are multipotent adult progenitor cells that have antitumor properties, and they can migrate to cancer cells and tumors. This study aimed to assess whether human adipose tissue-derived MSCs (hAD- MSCs) have the potential to deliver exogenous miRNAs to NB cells to induce differentiation and decrease proliferation of cancer cells.

**Materials and Methods:**

In this experimental study, hAD-MSCs were isolated, cultured, and differentiated. The M17 human NB cell line were also cultured. A specific type of miRNAs, i.e., miR-124 was successfully delivered to M17 NB cells with the aid of hAD-MSCs using the direct or indirect (exosome-based) contacts.

**Results:**

It was shown that indirect delivery of miR-124 considerably decreased the proliferation of NB cells and induced their differentiation.

**Conclusion:**

The results suggest the use of delivered exogenous miRNAs by the derived exosomes from hAD-MSCs as a novel cell-free stem cell-based therapy for NB cancer.

## Introduction

Neuroblastoma (NB) is one of the frequently observed malignant solid tumors in children that accounts for nearly 7% of childhood cancers and more than 15% of pediatric cancer deaths. The most commonly used treatments for metastatic NB are powerful chemotherapy or intensive chemotherapy with autologous hematopoietic rescue after the removal of the initial tumor (1). Despite the great advances made in multimodal treatments of NB, its prognosis in metastatic cases is still relatively poor. As widely accepted, NB is caused by imperfect differentiation of neural crest cell precursors of the sympathetic nervous system (2). Therefore, the approach of differentiation therapy could be the most appropriate and effective therapeutic option for NB. The ability of undifferentiated fatal cells in differentiation into mature cells results in arrest of cell growth and apoptosis (3). 13-cis-retinoic acid (RA), as a differentiation-inducing agent, is one of the standard mainstays of therapy in high-risk NB patients (4). This type of treatment leads to a significant increase in patient survival. However, at the same time, more than half of the treated patients develop recurrence. It must also be noted that RA therapy may result in the adverse effects of cell toxicity and inflammation. Therefore, the development of novel therapy for NB is an urgent issue.

MicroRNAs (miRNAs) are noncoding RNAs that play critical roles in the coordinated regulation of gene sets. Indeed, studies performed on the regulatory mechanism of miRNAs have attracted much attention in recent years. They can regulate different cellular processes, such as proliferation, differentiation, apoptosis, invasion, and angiogenesis (5). When miRNAs are expressed in specific tissue types, they can effectively contribute to differentiation. MiR-124 is a member of the nervous system-specific miRNAs regulating neurite outgrowth during neuronal differentiation. The level of miR-124 is increased during neuronal differentiation, and it plays an outstanding role in the development of neurons (6). Its overexpression in human glioblastoma multiforme cells induces neuronal phenotype (7). It is now known that the overexpression of miR-124 in stem cells leads to terminal neuronal differentiation with reduced malignancy (8). Despite the well-established therapeutic potential of miR- 124, its efficient delivery to the targeted tumor cells is a challenging task.

In recent years, studies suggested that mesenchymal stem cells (MSCs) have tropism to tumor sites and produce antitumor effects (9). They can also act as delivery vehicles for therapeutic miRNAs to transfer them into the region of cancer cells. The in vivo experiments indicated that MSCs promote NB differentiation and suppress tumor proliferation (10). As we reported previosly (11), MSCs derived from Wharton’s jelly have the capability to deliver miR-124 to glioblastoma multiforme cells and decrease cell migration and proliferation as well as indicing chemosensitivity. Based on the mentioned evidence, it can be expected that the use of MSCs for transferring miR-124 to cancer cells holds great promise for the treatment of NB.

In this study, we reported the transfer of miR-124 to NB cells with the aid of MSCs. It was shown that human adipose tissue-derived MSCs (hAD-MSCs) can deliver miR-124 to NB cells and this delivery system regulates the transcription profile and changes the function of cancer cells. It was demonstrated that indirect delivery of miR- 124 in the form of secreting exosomes from hAD-MSCs significantly decreases the proliferation of NB cells and induces their differentiation.

## Materials and Methods

In this section, the preparation of the cell samples and the methods used for characterizations are fully described.

### Ethical considerations

All procedures performed in studies involving human participants were in accordance with the ethical standards of the Research Ethics Committee of Tehran University of Medical Sciences (No. 92-01-87-21665-85626).

### Isolation, cell culture, and cell differentiations of human adipose tissue-derived mesenchymal stem cells

hAD-MSCs were obtained from healthy donors undergoing esthetic surgery. The isolation of hAD-MSCs was performed as descrived previosly (12). The digestion of adipose tissue was conducted at 37˚C with 1 mg/ml collagenase type I (Gibco, USA). After centrifugation of the suspension, hAD-MSCs were cultured in DMEM (Invitrogen, USA) supplemented with 10% fetal bovine serum (FBS, Gibco, USA), 100 g/ml streptomycin (Invitrogen, USA), 100 U/ml penicillin (Invitrogen, USA), and 2 mM L-glutamine (Invitrogen, USA). Replacement of the medium of the cultured cells and removal of the non-adherent cells were carried out after 48 hours. After 3 weeks, detachment of hAD-MSCs was performed when the cells reached 70-80% confluence. hAD-MSCs were characterized by the positive expression of CD73, CD105, and CD90 (Abcam, UK) markers and the negative expression of hematopoietic stem cell markers, namely HLA-DR (Abcam, UK), CD34 and CD45 (PE, eBiosciences). In order to study the multipotential differentiation of cells, particular cell culture media were utilized for the induction of the differentiation of hADMSCs into osteocytes and adipocytes. Dexamethasone (10 M) and insulin (6 ng/ml) were added to the cell culture medium to induce adipogenic differentiation in the plated cells. Similarly, for osteogenic differentiation, ascorbic acid (50 μg/ml), dexamethasone (10 M) and sodium β-glycerophosphate (10 mM) were applied. After 3 weeks, the plates were washed, and the cells were stained with Alizarin Red (13) and Oil Red O (14) to confirm their osteogenic and adipogenic differentiation, respectively.

### The cell culture of M17 cell line

In this experimental study, the cloned M17 human NB cell lines derived from the SK-N-Be (2) NB cell line were used (ATCC manassas, VA). HEK T293 and human M17 NB cell lines were purchased from the Pasteur Institute of Iran.

M17 cells were cultured in the culture medium containing a mixture of DMEM and F12 medium at a ratio of 50:50 supplemented (to final concentration) with 10% FBS, 100 U/mL penicillin, 0.1 mg/mL streptomycin, 2 mM L-glutamine and 1% nonessential amino acids (Invitrogen, USA) (15).

### miR‑124 Transfection

Cy3 (Life Technologies, Invitrogen, USA) was used for labeling RNA duplexes corresponding to hasmiR- 124. The transfections of hAD-MSCs, at passeges 3-4, was conducted using the Lipofectamine 3000 kit (LifeTechnologies, Inc., Invitrogen, USA) according to the manufacturer’s instructions (16).

### Preparation of exosomes

The cell culture of the transfected hAD-MSCs with miR-124-Cy3 and control miR was performed in the MSC medium using Gibco™ Exosome-Depleted FBS (Thermo Fisher Scientific, USA). After incubating for four days, the isolation of the exosomes from the supernatants of the hAD-MSC culture medium was carried out using the exosome precipitation solution, ExoQuick (System Biosciences). The protein content was evaluated by the Micro BCA assay kit (Sigma-Aldrich, Sweden) (17).

### Co‑culturing human adipose tissue-derived mesenchymal stem cells with M17 NB Cells

Following the manufacturer’s protocols, M17 NB cells were labeled with green fluorescence CMFDA Cell Tracker (Molecular Probes). After labeling the cells, they were mixed at a ratio of 50:100 by hAD-MSCs transfected with miR-124 and plated in 8-well plates. After 72 hours, flow cytometric analysis was performed for confirming the delivery of miR-124 to M17 cells.

To check the delivery of miR-124 mimetic with an indirect contact (i.e., via the secreted exosomes from hADMSCs), membranes with 0.4 μm-pore diameter were used in a transwell chamber to inhibit cell infiltration (hADMSCs- miR-124-Cy3 and M17 cells). Plating hAD-MSCs transfected with with miR-124-Cy3 was carried out in the transwell inserts. At the same time, seeding M17 NB cells was performed in the lower well. After 72 hours, M17 NB cells were collected, and the flow cytometric analysis was applied to ensure that miR-124-Cy3 was delivered to M17 cells.

### Quantitative real-time polymerase chain reaction

The QIAzol reagent (Qiagen, Germany) was used for the extraction of the total RNA. The synthesis of complementary DNA (cDNA) was also performed by reverse transcriptase (Fermentas, Germany) and random hexamers for gene primers. Triplicate real-time polymerase chain reaction analysis with SYBR Premix Dimer EraserTM (TaKaRa, Japan) was used, and the results were analyzed using the REST and the Rotor-Gene 6.1 (Corbett, Australia) software. The PCR primers and their respective reverse complements were as follows:

*h-Tubullin beta III*-

F: 5´-GGA GTA TTT GGA TGA CAG AAA C-3´

R: 5´-GAT TAC CAC TGG AGT CTT C-3´ (product length: 238 bps)

MAP2-

F: 5´-AGT TCC AGC AGC GTG ATG-3´(product length: 164 bps)

R: 5´-TAGTCTAAGCTTAGC TGAGAATCTACCGA-3´

GAPDH

F: 5´-GAC AAG CTT CCC GTT CTC AG-3´

R: 5´-GAG TCA ACG GAT -TTG GTC GT-3´ (product length: 132 bps).

*GAPDH* mRNA was used as the internal control. The real-time PCR protocol was as follows: 2 minutes at 95˚C, 5 seconds at 95˚C for denaturation, 30 seconds at 60˚C for annealing, 10 seconds at 72˚C for amplification, and 40 cycles of extension (18).

### Cell viability and apoptosis assay

In order to investigate the cell viability, M17 cells were seeded at a density of 5×10^3^ in 96-well plates and incubated overnight while keeping the temperature constant at 37˚C. The addition of the exosomes derived from control-miR and hAD-MSC-miR-124-Cy3 to M17 cells was carried out after 24 hours. The flow cytometry analysis was performed to detect the delivery of exosomes containing miR-124. Upon the assurance of the delivery of miR-124 to the M17 cells, the viability of M17 cells transfected with miR-124 and control miR was examined by the MTT assay after 24, 48 and 72 hours. The rate of apoptosis in M17 cells transfected with miR-124 and control miR was assayed using terminal deoxynucleotidyltransferase dUTP nick-end labeling (TUNEL) assay performed by the In Situ Death Detection kit (Roche Diagnostics, Indianapolis, IN) according to the manufacturer’s instructions. The cultured M17 NB cells with the secreted exosomes from hAD-MSCs-Con-miR (M17-hAD-MSCs-Con-miR) were treated with derived exosomes from hAD-MSCs-miR-124 (M17-hAD-MSCsmiR- 124). The cultured M17 NB cells were also directly transfected with miR-124 (M17-miR-124) and its control (M17-con-mir).

In this study, the data were analyzed by the Statistical Package for Social Sciences version 11 software (SPSS, IBM, USA).

## Results

**Fig.1:**
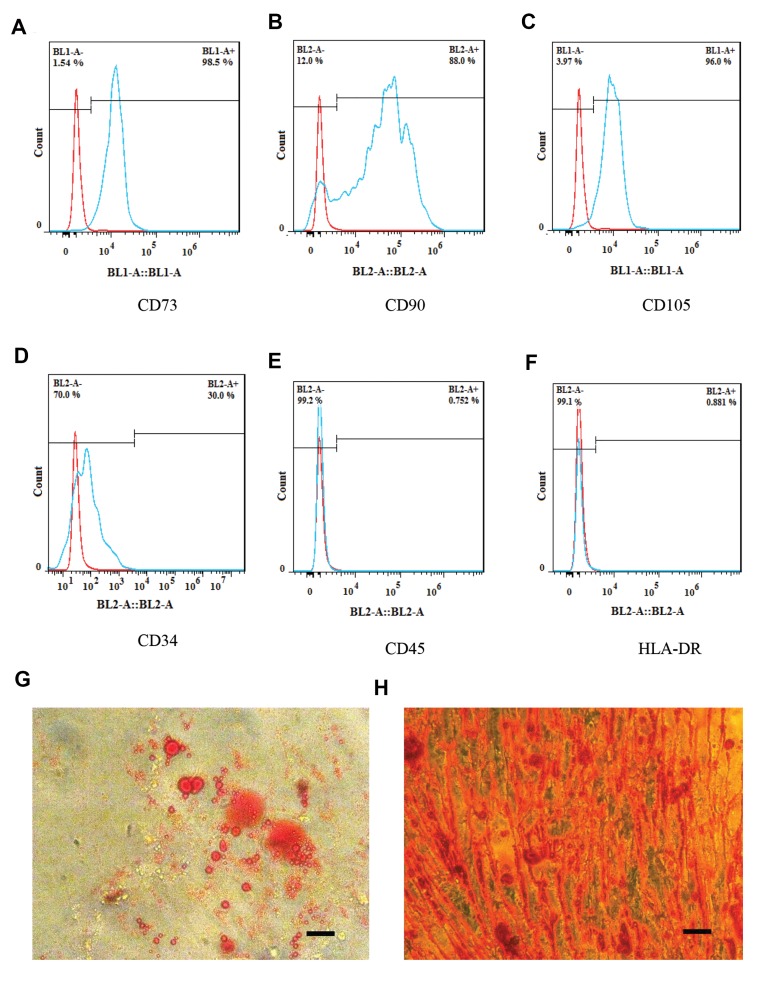
Characterization of human adipose tissue-derived mesenchymal stem cells (hAD-MSCs) by flow cytometry and light microscopy. The flow cytometry analysis of hAD-MSCs for the detction of **A**. CD73,** B**. CD90,** C**. CD105 (positive markers), **D**. CD34,** E**. CD45, **F**. HLA-DR (negative markers). Light microscopy images show **G**. Adipogenic and **H**. Osteogenic differentiation of hAD-MSCs (scale bar: 200 μM).

**Fig.2:**
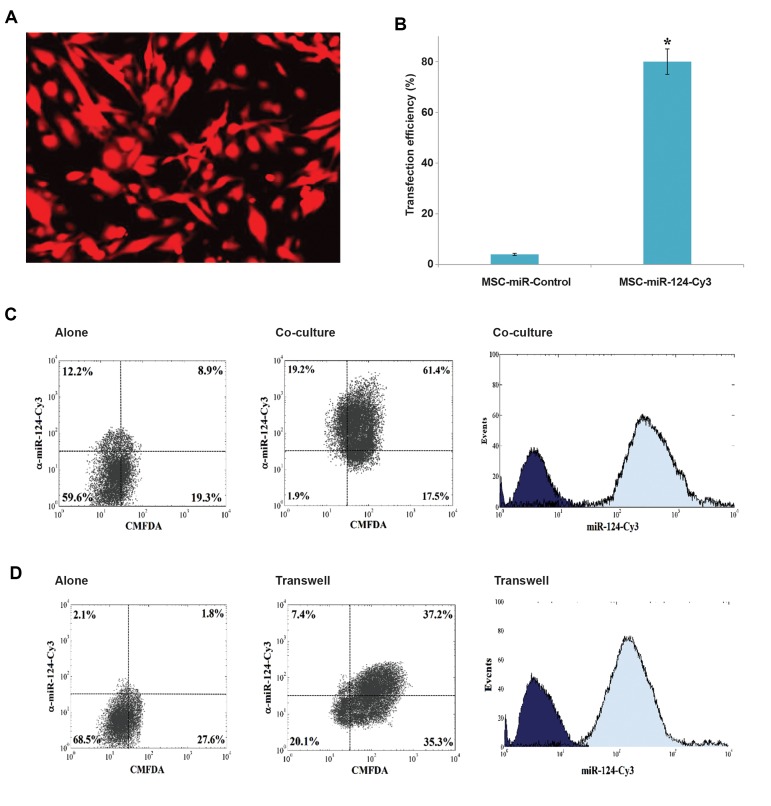
The results of flow cytometry confirmed the delivery of miR-124 to M17 NB cells by human adipose tissue-derived mesenchymal stem cells (hADMSCs). HAD-MSCs were transfected with Cy3-labeled miR-124. After 24 hours, the labeled M17 NB cells with Green Cell Tracker CMFDA were added to the ceulture medium of hAD-MSCs. The expression of the fluorescent miR-124 in M17 NB cells was analyzed after 24 hours by flow cytometry. The results indicated the transfer of miR-124-Cy3 from hAD-MSCs into M17-CMFDA cells.** A**. hAD-MSCs were transfected with cy3-lablled miR-124. **B**. Transfection efficiency was estimated about 80% by fluorescence microscopy (P<0.05). **C**. M17 NB cells co-cultured with hAD-MSCs-miR-124-Cy3, left panel: NB cells alone; middle panel: analysis of NB cells and hAD-MSCs for CMFDA and Cy3; right panel: Cy3 in co-cultured cells. **D**. Exosomes derived from hAD-MSCs were added to NB cells with transwell, left panel: NB cells alone; middle panel: the analysis of M17 NB cells for CMFDA and Cy3; right panel: Cy3 alone in M17 NB cells. *; P<0.05.

As shown in Figures 1A-C, the flow cytometry analysis indicated the expression of CD73, CD90, and CD105 in hAD-MSCs. Furthermore, the cells were negative for HLA-DR, CD34, and CD45 (Fig.1D-F). The multipotency of hAD-MSCs was also confirmed by adipogenic and osteogenic differentiation (Fig.1G, H). Transfection efficiency was estimated about 80% by fluorescence microscopy (Fig.2A, B). In the following sections, the delivery of hAD-MSCs to M17 NB cells and their subsequent effects on inducing apoptosis and neuronal differentiation in NB cells is indicated.

### Delivery of miR-124 mimetic to M17 NB cells

According to previous reports (19, 20), MSCs have the ability of cell-to-cell communication via gapjunctional intercellular channels (direct contact) or by secreting different factors, such as cytokines, vesicles, and extracellular matrix molecules (indirect contact) that promote neurogenesis. Furthermore, MSCs can also be genetically modified to be able to release specific growth factors, cytokines, and miRNAs in the form of exosomes (21). In this paper, the potential of hAD-MSCs to deliver exogenous miRNA mimetics to M17 NB cells was examined. Specifically, the focus of this study was on miR-124 delivery because this miRNA has already been reported to have a significant role in differentiation of NB cells (8). For this aim, two days after the co-culture period of hAD-MSCsmiR- 124 with M17 NB cells (direct contact), the combination was studied with the aid of the twochannel flow cytometry technique. The direct transfer of miR-124 from hAD-MSCs-miR-124 into M17 NB cells was confirmed by the detection of Cy3 in M17 NB cells (Fig.2C). Moreover, in transwell-cultured hADMSCs, the detection of miR-124-Cy3 indicated that M17 cells were Cy3-positive, and hence, miR-124-Cy3 was indirectly transferred from hAD-MSCs into M17 NB cells (Fig.2D). Figure 2C corroborates that miR-124 was indirectly transferred with exosomes derived from hADMSCs into M17 NB cells. The bi-color flow cytometry dot plots in Figure 2 represent the percentage of the coand the transwell-cultured cells.

### Concomitant decreased proliferation and apoptosis induction in M17 NB cells through the delivery of miR-124 by hAD-MSCs

**Fig.3:**
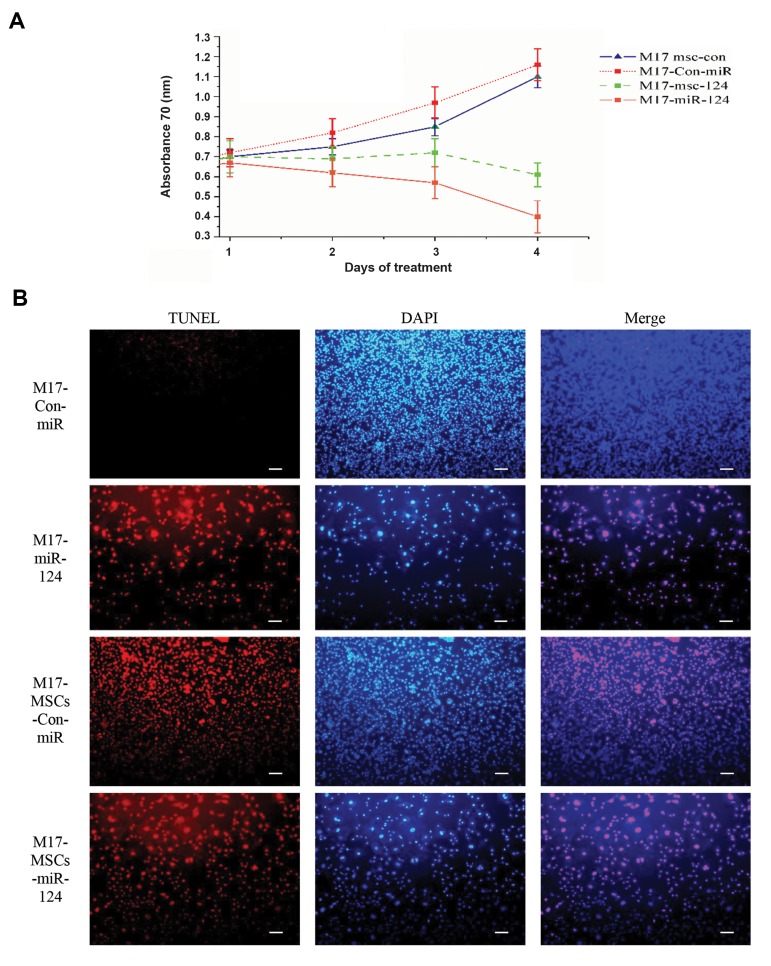
The results of inducing differentiation in M17 NB cells after delivery of miR-124 by hAD-MSCs. **A**. The MTT assay represents the cell proliferation in miR-124-treated cells in every 24 h interval and **B**. TUNEL staining results indicating that the delivered miR-124 induced considerable apoptosis 24 hours after exposure. The nuclei of TUNEL-positive cells show that most of the cells underwent apoptosis. The cultured M17 NB cells with exosomes secreted from hAD-MSCs-Con-miR (M17-hAD-MSCs-Con-miR) were treated with exosomes derived from hAD-MSCs-miR-124 (M17-hAD-MSCs- miR-124). The directly transfected M17 NB cells with miR-124 (M17-miR-124) and its control (scale bar: 50 μM).

To determine whether transferring miR-124 to M17 NB cells by hAD-MSCs may have an effect on proliferation in addition to induction of apoptosis, the derived exosomes from hAD-MSC-miR-124 cells were used for treating M17 cells. The result of the MTT assay showed that the delivery of miR-124 reduced the proliferation of M17 cells (Fig.3A). In order to further check whether the delivery of miR-124 can induce apoptosis in M17 NB cells, the TUNEL assay was carried out, as well. As indicated in Figure 3B, it was demonstrated that miR-124 induced apoptosis in M17 NB cells.

### MiR-124 mimetic delivery by hAD-MSCs stimulates the neuronal differentiation of M17 NB cells

**Fig.4:**
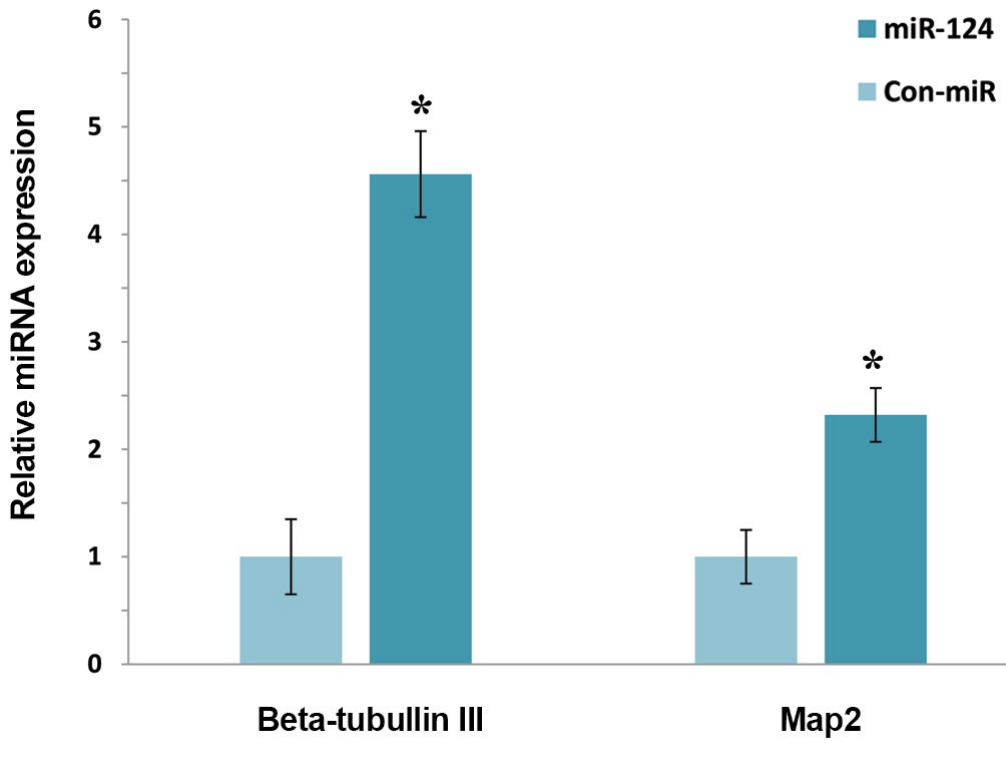
The qRT-PCR analysis results, showing the increase of mRNA level of b-Tubulin III and MAP2 (P<0.05). The results indicate that hAD-MSCs delivered miR-124 and induced differentiation in M17 NB cells in comparison with the control cells. *; P<0.05 and qRT-PCR; Real-time quantitative reverse transcription polymerase chain reaction.

As mentioned earlier in previous sections, the sole delivery of miR-124 to NB cells without any intermediate element has been reported to induce differentiation in cells (8, 22). The goal of this study was to examine the role of MSCs, as intermediate carriers, in facilitatating the delivery of miR-124 to M17 NB cells and inducing further differentiation. In order to investigate this, M17 cells were treated with the exosomes derived from hAD-MSCs-miR-124 and showed that the expression of b-tubulin III and MAP2 significantly enhanced in comparison with the control (Fig.4). This results confirm that miR-124 delivery induced the differentiation of M17 NB cells. Additionally, as depicted in Figure 3, M17 cells treated with exosomes derived from hAD-MSCs-ConmiR also induced cell differentiation. In the same manner, the induction of differentiation was rather spectacular in M17 cells treated with exosomes secreted from hADMSCs- miR-124. The increased differentiation in this case can be ascribed to synergistic effect of neurotropic factors secreted from both hAD-MSCs and miR-124. Furthermore, compared with the undifferentiated cells, the morphology of neuron-like cells was more pronounced in differentiated M17 cells . This is consistent with previous findings that the overexpression of miR-124 noticeably induces differentiation of NB cancer cells (8, 22).

## Discussion

Recently, miRNAs have been appeared as the main potential therapeutic targets in cancers (23) and expression alternation of miRNAs in different neurological disorders have been the subject of several studies (24, 25). Indeed, it is already shown that administration of miR-based therapy will provide therapeutic approaches in pathological conditions of the central nervous system cancers (26).

However, despite their therapeutic potential, the existing problems in the way of controlled delivery of therapeutic agents to targeted neural cells are mostly considered as the major reasons for the poor outcomes of treatment.

In regenerative medicine, MSCs are known to be hopeful sources for cell therapy since they have immunomodulation, trophic factor secretion and transdifferentiation properties (27). Furthermore, the secreted exosomes from MSCs that assist in restraining tissue injuries, lead to re-entry of cell cycle in resident cells and induce tissue self-repair, are being examined for various applications in neural, musculoskeletal or cardiac repair (28). In recent years, various studies have indicated that MSCs can be effectively used for treating different disorders in the central nervous system, including Parkinson’s disease, multiple sclerosis, amyotrophic lateral sclerosis (ALS) and stroke (29, 30). MSCs show tropism to malignant cells, migrate to tumor microenvironments, exert antitumor effects and also have this ability to act as delivery vectors for anticancer agents (31). MSCs and neural stem cells are also considered as promising candidates for overcoming the blood-brain barrier by delivering drugs and RNAs to tumours or neurodegenerative disorders (32).

In this study, the ability of hAD-MSCs to act as a delivery vector for transferring miR-124 to co-cultured NB cancer cells has been demonstrated, and they are proposed as a promising approach for the targeted delivery of miRNA-based therapy to NB cancer cells. Previously, Bianchi et al. reported that functional crosstalks between hMSCs and NB cell lines can be effective only within short range interaction and showed that intravenously inoculated hMSCs in different NB models did not reach the tumor sites (10). Nevertheless, they also showed that intratumorally injected hMSCs in a subcutaneous NB model decreased tumor growth and enhanced the survival time. On the contrary, Kimura et al. have reported that intraperitoneally administered hMSCs can migrate and affect tumor cells in a TH-MYCN mouse model (33). In a recent clinical study, the efficiency of the inhibition of bone marrow metastasis in NB was shown by menas of the infected autologous MSCs with ICOVIR-5. Excellent treatment tolerance and full clinical response were reported for this new type of treatment (34). It was also shown in our previous work (11) that MSCs of Wharton’s jelly deliver exogenous miRNAs to glioblastoma multiform cells and their functional effects were fully elucidated. It has been demonstrated that the labeled miR-124 can be delivered effectively by MSCs of Wharton’s jelly to U87 glioblastoma multiform cells through exosome-dependent or independent manners. Consistent with these reports, in this study, it was also found that hAD-MSCs can successfully deliver miR-124 to the co-cultured M17 NB cells by localizing the Cy3- labeled miR-124. Furthermore, it was revealed that miR- 124 delivery by exsosomes secreted from hAD-MSCs to M17 NB cells holds great promise for delivery of miRNAs to target cells.

It is now know that miR-124 is a neuron-specific miRNA that has a great impact on neurogenesis and differentiation of neuronal cells (35). According to previous reports, miR-124 has the ability to act as proliferation inhibitor, and thereby the suppression of CDk6, can induce cell differentiation (36, 37). Also shown in our previous work, the overexpression of miR-124 increased the the expression levels of MAP2, b-Tubulin III, NF-M, SYN and Nestin markers and induced the functional differentiation in M17 NB cells (22). In this study, it was indicated that exosomes secreted from hAD-MSCsmiR- 124 promotes miR-124 delivery to M17 NB cells and reduces their proliferation. The proliferation control is a critical step in terminal differentiation program in tumor cells (38). Therefore, in the current work, after successful delivery of miR-124 with exosomes secretd from hAD-MSCs to the M17 NB cells and approving their proliferation decrease, the induction of differentiation in these cells was also investigated. The obtained results confirmed that exosome delivery of miR-124 to M17 NB cancer cells can be an efficient cell-free approach for differentiation of NB cancer. In a previously published report, it was demonsrtrated that secreted neurotrophic factors from MSCs can solely induce differentiation in neuronal progenitor cells (39). Furthermore, exosome secreted from MSCs, as a novel cell-free stem cell-based therapy and the genetically modified exosomes, have yielded positive therapeutic results (40). In the present study, it was shown that the induction of differentiation in the M17 NB cells treated with exosomes secreting from hAD-MSCs-miR-124, compared with the control (M17 NB cells treated with exosomes secreted from hAD-MSCs-Con-miR), is the outcome of two distinct factors, i.e., miR-124 and hAD-MSCs. Considering these results, using hAD-MSCs as a vector for miR-124 delivery to NB cells seems very useful for the treatment of NB cancer.

## Conclusion

In this study, it was demonstrated hAD-MSCs can efficiently deliver exogenous miR-124 to NB cells which, in turn, decrease their proliferation and stimulate the induction of their differentiation. The obtained results suggest the opportunity to use the delivered exogenous miRNAs by hAD-MSCs, as a novel cell-free stem cellbased therapy, for the treatment of NB cancer. Future studies can be directed towards more investigations on allogeneic MSCs by murine tumor models, which are necessary for the confirmation of the antitumor potential of MSCs.
